# Methods for RNA Modification Mapping Using Deep Sequencing: Established and New Emerging Technologies

**DOI:** 10.3390/genes10010035

**Published:** 2019-01-09

**Authors:** Yuri Motorin, Mark Helm

**Affiliations:** 1 Laboratoire IMoPA, UMR7365 National Centre for Scientific Research (CNRS)-Lorraine University, Biopôle, 9 Avenue de la Forêt de Haye, 54505 Vandoeuvre-les-Nancy, France; 2 Institute of Pharmacy and Biochemistry, Johannes Gutenberg-University Mainz, Staudingerweg 5, 55128 Mainz, Germany

**Keywords:** RNA modification, epitranscriptome, deep sequencing, Next Generation Sequencing, RNA-Seq, chemical treatment, engineered Reverse Transcriptase enzymes

## Abstract

New analytics of post-transcriptional RNA modifications have paved the way for a tremendous upswing of the biological and biomedical research in this field. This especially applies to methods that included RNA-Seq techniques, and which typically result in what is termed global scale modification mapping. In this process, positions inside a cell’s transcriptome are receiving a status of potential modification sites (so called modification calling), typically based on a score of some kind that issues from the particular method applied. The resulting data are thought to represent information that goes beyond what is contained in typical transcriptome data, and hence the field has taken to use the term “epitranscriptome”. Due to the high rate of newly published mapping techniques, a significant number of chemically distinct RNA modifications have become amenable to mapping, albeit with variegated accuracy and precision, depending on the nature of the technique. This review gives a brief overview of known techniques, and how they were applied to modification calling.

## 1. Introduction

Precise and reliable detection of the numerous modified nucleotides in RNA is still a highly challenging task in the epitranscriptomics field. In addition to traditional detection and quantification strategies, including various types of chromatography such as TLC, HPLC, and/or LC-MS(MS), approaches relying on deep sequencing have emerged in force, and numerous applications to large transcriptomes were reported, starting in 2012 [1,2,3,4,5], with a particularly dynamic development during the past two–three years. These methods provided an unprecedented amount of information and depth, in certain cases even allowing the analysis of low abundant RNA species. In this brief overview, we consider techniques that utilize deep sequencing for modification calling, including both well-established and innovative methods. The current portfolio of concepts for the selective detection of RNA modifications ranges from antibody-(Ab)-based anti-methylated/modified nucleoside immunoprecipitation (MeRIP) and variants of individual-nucleotide-resolution crosslinking and immunoprecipitation (iCLIP) method (also called miCLIP) [6,7], over the use of particular enzymes conferring specificity, to applications of known and newly developed organic chemistry of nucleic acids.

## 2. Antibody-Based Technologies (MeRIP-Seq, i/miCLIP)

The first application of m^6^A-Ab-based enrichment dates back to the 1980s [8], but only its coupling to deep sequencing brought on the revolution in the field that we are seeing today. The elucidation of human and mouse m^6^A-methylomes by MeRIP-Seq immunocapturing and sequencing [2,9], was, and still is, being followed by a vast number of studies using this technology, or a derivative thereof. Indeed, despite many openly discussed shortcomings of the various available anti-m^6^A antibodies, mapping of this modification, especially in mRNA, is arguably the most frequent and visible application in the field. The initial embodiments of MeRIP-Seq had only low resolution and provided broad peaks of ~100–200 nt in which the actual m^6^A modification site was supposed to be contained. Further improvements of this technique came from the combination of MeRIP with Ab cross-linking, allowing single nucleotide resolution. The latter technique is a specialized derivative of CLIP, termed miCLIP (methylation individual-nucleotide resolution UV cross-linking and immunoprecipitation, [6]).

Thus far, MeRIP-Seq or Ab-related techniques were also applied to the detection of hm^5^C [10], ac^4^C [11,12], m^5^C [13], and of m^1^A [14,15,16,17], even though m^1^A data remain highly controversial [18,19]. Despite their massive use in different studies, all Ab-related approaches suffer from poorly characterized, and thus unpredictable specificity of the Ab used for enrichment [20]. These uncertain biases may include sequence context, nucleobase specificity, and a presumed cooperativity in the binding to multiple methylation sites in the same fragment (discussed in Reference [21]), plausibly caused by the presence of two F_ab_ copies in a typical IgG. In addition, the exact stoichiometry of the modification remains largely unknown, since peak enrichment produces signals whose intensities are not likely to be linearly correlated to the original level of modification. More precise quantification was introduced by LAIC-Seq [22], where both m^6^A-positive and negative mRNA fractions from the same sample were converted to libraries and sequenced in parallel. Careful calibration with spiked-in mRNA standard allows more exact approximation of the m^6^A stoichiometry in mRNAs. Data obtained in two human cell lines showed that molar modification levels varied considerably from one mRNA site to another, and covered the entire range from almost zero to 1 mol m^6^A/mol mRNA. 

As mentioned above, miCLIP applications to MeRIP-Seq brought considerable improvement, allowing detection via analysis of reverse transcriptase (RT)-stops and/or misincorporations, which occur as a consequence of the cross-link events either at the m^6^A position or at the +1 site [7,23]. Similarly, m^6^Am, which is frequently present at m^7^G-mRNA cap, was detected in the form of a mutational signature [7]. Overall, miCLIP is thought to provide better specificity in mapping, since the simultaneous presences of an enrichment peak and the RT-signature improve confidence in modification calling. Another variant of miCLIP (PA-m^6^A-Seq) used s^4^U incorporation for PhotoActivatable-Ribonucleoside-Enhanced Cross-Linking and Immunoprecipitation (PAR-CLIP, [24]). 

## 3. Detection Using Natural or Induced RT-Arrests and Signatures

### 3.1. Naturally Existing RT-Signatures

Modifications in an RNA template are known to potentially cause reverse transcriptases to either arrest, to incorporate a dNTP cognate to the unmodified template, or to misincorporate non-Watson-Crick compatible dNTPs into the nascent cDNA. The combination of these events constitutes the so-called RT-signature, which varies with the type of modification, and likely with reaction conditions including the type of enzyme used [25]. One of the first attempts to use RT-misincorporation events caused by modifications in the RNA template was termed HAMR for High-throughput Annotation of Modified Ribonucleotides [26,27]. This approach was used to map some potential m^3^C, m^1^A, m^1^I, m^2^_2_G, and m^1^G sites in human tRNAs, of which a selection of predicted m^3^C sites were experimentally validated. 

The idea of a specific RT-signature was developed in application to m^1^A detection in rRNA and tRNAs [25,28]. It was demonstrated that an RT-signature is sequence context dependent, but may be used for mapping of unknown modification sites, and, after thorough calibration, even for their relative quantification.

More recently, the analysis of m^1^A-generated RT-signatures was extended to analysis of the human transcriptome [17]. Non-enriched mRNA samples show only a very few high-confidence sites with m^1^A-induced RT-signature, but this level can be increased by prior MeRIP type enrichment with anti-m^1^A-Ab, indicating that modification levels at the relevant sites were most probably sub-stoichiometric.

The detection of modifications via their RT-signature can only be applied to a limited subset of known RNA modifications, since many do not affect Watson-Crick base pairing during cDNA synthesis and thus are RT-silent. Interestingly, for some presumed RT-silent modifications, particular conditions were identified in which they become visible [29,30]. For example, it was observed early on that ribose 2′-O-methylations in RNA could be detected as RT-stops at reduced dNTP concentrations (1–5 µM final concentration) during primer extension. This method was extensively used in the past for mapping of 2′-O-Me groups in various rRNAs [31]. This methodology was also coupled to NGS for mapping of 2′-O-Me [32], termed 2OMe-seq. Applications of this techniques were only described for validation of methylation sites in rRNA, since RT-primer extension at low [dNTP] is rather prone to generate false-positive hits. In addition, the intensity of RT-stop varies for different sequence contexts, leading to uncertainties in quantification.

### 3.2. Enhancing RT-Signatures with Engineered Enzymes and Substrates

**Figure 1:**
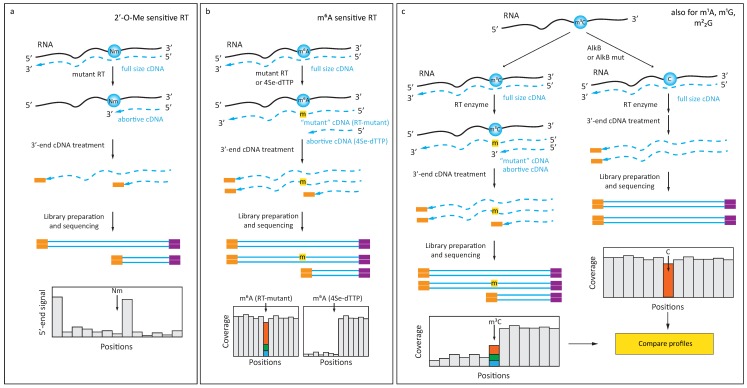
Detection of 2′-O-methylation, m^6^A and AlkB-sensitive modifications in RNA (**a**) Engineered polymerase arrests reverse transcription at 2′-O-methylated residue at normal [dNTP]; (**b**) Ab-independent detection of m^6^A residue using an engineered polymerase or a 4-Se derivative of dTTP (4Se-dTTP). The presence of an m^6^A residue in the RNA template is reflected by a misincorporation signature in the cDNA; (**c**) Improved confidence in the detection of AlkB-sensitive RNA modifications (m^3^C, m^1^A, m^1^G, and m^2^_2_G) comes from comparison of RT-signatures (including misincorporation and RT-arrest) before and after AlkB treatment.

In addition to manipulating reaction conditions during RT, RT-silent RNA modifications can also be detected using particular, notably engineered RT-enzymes. A first example was an engineered version of a thermostable KlenTaq DNA polymerase, which displayed significant intrinsic RT activity. Mutant KlenTaq DNA polymerases were generated that displayed sensitivity to 2′-O-Me RNA residues even at normal dNTP concentrations (Figure 1a), and these were used for RTL-P-like [33] detection of modifications [34]. Applications of this enzyme to high throughput RNA-Seq have not yet been described, but appear promising in 2′-O-methylation detection. Additionally, starting from a KlenTaq DNA polymerase, protein engineering yielded an enzyme sensitive to m^6^A (Figure 1b), which exhibits increased misincorporation at respective sites of m^6^A in the template [35]. This enzyme, too, still awaits application to transcriptome wide analysis.

On the other hand, chemical manipulation at the level of the dNTPs used for primer extension can also exacerbate the RT-signature for specific RNA modifications (Figure 1b). One specific dNTP featuring a selenium substitution of the O4 of thymidine (4Se-dTTP) was found to induce RT-stops and used to generate a specific signature for m^6^A [36]. Combined with FTO-assisted RNA de-methylation, this approach may potentially become an alternative to Ab-dependent m^6^A detection strategies with single nucleotide resolution.

Finally, in addition to the manipulation of enzyme and dNTPs, modulation of an RT-signature can also be achieved by manipulation of the RNA template. Enzymatic removal of methyl groups by demethylase enzymes of the AlkB-type was demonstrated for m^1^A, m^3^C, and m^1^G RNA modifications, which are commonly found in tRNAs (Figure 1c). A coupling of this approach to deep sequencing was developed into a method termed ARM-Seq [37], which used pretreatment of RNA with *Escherichia coli* AlkB to remove RT-blocking modifications. Another method using the same basic idea, in which WT AlkB was combined with an AlkB D135S mutant of improved activity, was called DM-tRNA-Seq [38].

More recently, another AlkB variant (D135S/L118V) was shown to efficiently and selectively de-methylate m^2^_2_G in tRNAs, thus improving tRNA deep sequencing and allowing more specific detection [39].

## 4. Exploiting Specific Chemical Reactivity of Modified Nucleobases

### 4.1. Chemically Induced Alteration of RT-Profiles

The property of naturally occurring RNA modifications to affect the performance of RT enzymes during cDNA synthesis in terms of misincorporation or RT-arrest can be modulated by chemical treatment prior to the RT reaction. Various reagents are known to either selectively react with modified nucleosides, or to selectively affect non-modified nucleotides. An example for the latter is bisulfite sequencing, which was adapted from 5mC detection in DNA to application in RNA by Schäfer and Lyko [40]. The bisulfite reagent is a nucleophile that performs a Michael addition to cytidines, ultimately leading to their deamination. The methyl group in m^5^C is thought to increase the electron density of the nucleobase, thus leading to repulsion of the nucleophile and thus inertness of m^5^C to deamination under these specific conditions. Consequently, the readout consists in detection of cytidines in deep seq data sets, and is therefore, in principle, well suited for quantification and single nucleotide resolution mapping. The method is generally considered robust for abundant RNAs such as tRNA and rRNA [40,41,42,43], but results on lower abundant RNAs, including mRNAs and lncRNAs [4,13,44,45], are contested [46].

Further, building on bisulfite chemistry, procedures for mapping oxidation products of m^5^C were developed, which were, again, first applied on the respective DNA modifications. While hm^5^C yields signals that are indistinguishable from m^5^C in bisulfite sequencing, f^5^C remains invisible. However, the two species can be converted into each other by mild redox agents: f^5^C can be reduced to hm^5^C by NaBH_4_ (RedBS-Seq [47] in DNA, application to RNA: [48]), or be protected from conversion by treatment with *O*-ethylhydroxylamine [49], application to RNA [48]. Permutations of these treatments coupled to deep seq analysis have been used for mapping in both DNA and RNA. In DNA, hm^5^C was oxidized to f^5^C with K_2_RuO_4_, followed by an aldol-type addition-elimination-cyclization sequence [50], and the ensuing alteration of base-pairing properties was exploited for sequencing. However, this technique still awaits potential adaptation to RNA-seq.

**Figure 2:**
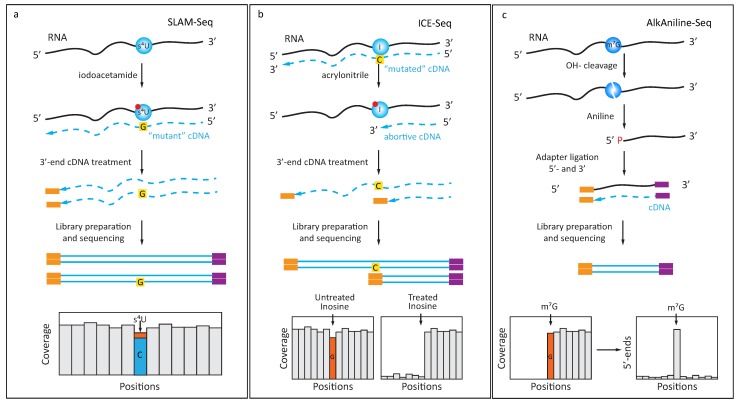
Use of specific chemical reagents for detection of s^4^U, inosine (I) and m^7^G/m^3^C: (**a**) 4-thiouridine (s^4^U) readily reacts with iodoacetamide, with subsequent change of base-paring properties. After iodoacetamide treatment, derivatized s^4^U is read as C during RT step and this U- > C transition is detected in the sequencing data; (**b**) Detection of inosine (I) in RNA by acrylonitrile derivatization (ICE-Seq). Unmodified inosine gives an A- > G misincorporation signature, which can be distinguished from SNPs by additional treatment with acrylonitrile. The resulting derivatized inosine creates abortive products during RT-primer extension; (**c**) AlkAniline-Seq protocol for detection of m^7^G and m^3^C residues in RNA. Mild alkaline hydrolysis creates an RNA abasic site at the sensitive residue and subsequent aniline treatment releases 5′-phosphate at the N + 1 nucleotide. These 5′-phosphates are used for selective adapter ligation during library preparation step. AlkAniline-Seq signal is detected as a substantial proportion of sequencing reads starting at the same position.

From PAR-CLIP experiments [51], it was known that the reverse transcription profile of s^4^U displays a certain percentage of misincorporation resulting in an U- > C transition in the cDNA. SLAM-seq [52] exploits an altered hydrogen binding pattern of s^4^U after alkylation with iodoacetamide (ICH_2_-CONH_2_), which causes s^4^U to be retrotrancribed as a C, increasing the apparent transition rate to near-quantitative (Figure 2a). While this method was mostly applied to trace the incorporation of s^4^U nucleoside into nascent RNA in metabolic labeling experiments, it is equally suited to trace posttranscriptional s^4^U modifications that occur, e.g., at position 8 of bacterial tRNAs.

Inversely, inosine, which is read like a guanosine by known RT-enzymes, does not normally display any tendency to cause RT-arrest. Treatment with acrylonitrile was shown to chemically modify inosines by alkylation, leading to RT-arrest at inosine sites (Figure 2b), and ultimately allowing identification of inosines in a transcriptome-wide search with increased confidence (ICE-Seq [53]).

In contrast, detection of pseudouridine must, so far, entirely rely on chemical treatment for the generation of RT-stops, as the nucleoside itself leaves no exploitable RT-signature. To this end, several labs have performed mapping experiments using soluble carbodiimide (*N*-Cyclohexyl-*N*’-(2-morpholinoethyl)carbodiimide metho-*p*-toluenesulfonate, usually abbreviated as CMCT) [3,54,55,56,57,58], an agent developed by Bakin and Ofengand [59] for mapping of pseudouridine in ribosomal RNA. A promising variant used a “clickable” derivative, which was conjugated with an affinity tag for the enrichment of derivatized, pseudouridine containing RNA [60,61]. Open questions include the moderate overlap of pseudouridine sites from the different studies [62], potentially a consequence of variable sequencing depth [63].

The reverse transcription signature of an RNA modification is, in principle, understood to also contain abortive RT events resulting in truncated cDNA, for which library preparation protocols must be specifically tailored. The composite nature of the RT-signature of m^1^A, which was already mentioned [25], can be specifically altered by alkaline treatment, causing a Dimroth rearrangement of m^1^A, whose product m^6^A does not display any significant signature. This reaction was used as a means of validation, whereby the RT-signature of m^1^A was altered by incubation at high temperature under alkaline conditions, leading to the m^1^A- > m^6^A conversion [14,17]. The resulting disappearance of m^1^A signals would be expected to coincide with the appearance of an m^6^A signal in, e.g., MeRIP-Seq data. Since the alkaline treatment causes significant RNA degradation, and because m^6^A detection is still inaccurate, applications of the Dimroth rearrangement so far have been limited to validation of predicted m^1^A sites, rather than for their global profiling.

One of the oldest combination of chemical treatments known to specifically react with a modified nucleotide [30,64] has only recently been combined with an RNA-Seq approach. Reduction of m^7^G with NaBH_4_ renders the corresponding site in the RNA chain susceptible to aniline induced cleavage, which was then analyzed as RT-stop in RNA-Seq data in a combination termed TRAC-seq [65].

A chemically related treatment is the reduction of ac^4^C with NaBH_4_, leading to saturation of the 5,6 double-bond in acetylated cytidines. This, in turn, produced ~20–30% of misincorporation signals in cDNA, as determined by Sanger sequencing [66]. This method, which still awaits its application to RNA-Seq, might profit from validation of acetylated sites by chemical deacetylation with hydroxylamine, which would be expected to erase signals [11].

### 4.2. Protection of RNA from Cleavage

Variations of the so-called RiboMethSeq approach, first published by the Nielsen lab and further independently developed by two other groups [67,68,69,70], are based on relative protection of phosphodiester bond in RNA when 5′-neighboring ribose is 2′-O-methylated. This protection can be converted into a signal after random alkaline hydrolysis, conversion of the resulting fragments to the sequencing library, and counting of 5′- and 3′-extremities after alignment to the reference sequence. Modern variants of RiboMethSeq require very low quantities of input RNA (10 ng is generally sufficient), thus opening the possibility for large-scale biomedical applications on clinical samples. Compared to other methods for 2′-O-Me detection (2′OMe-seq [32] and Nm(RibOxi)-Seq [71,72]), RiboMethSeq requires higher sequencing depth (minimum 100 reads/nt position in reference sequence), but provides precise site-by-site quantification of RNA modification, which is difficult to achieve by other approaches. This requirement for significant sequencing depth makes RiboMethSeq less appropriate for low abundant RNAs, but this limitation can be overcome by preliminary enrichment of target RNA or simply by increased sequencing depth for analysis [73].

### 4.3. Specific Enrichment of RNA Fragments by Selective Ligation

A novel trend in the field is the development of library preparation protocols which selectively produce RNA fragments with modified nucleotides at the 5′- or 3′-end extremity, properties which can then be exploited for enrichment of these fragments in RNA Seq libraries. Two recently developed methods, Nm-Seq [71] and RibOxi-Seq [72], exploit the stability of 2′-O-Me riboses against NaIO_4_ oxidation, under conditions where the unmodified *cis*-diol of a normal ribose is rapidly oxidized and cleaved, thus making it inaccessible to 3′-adapter ligation. In this protocol, RNA was randomly cleaved by nuclease to release 5′-phosphates and 3′-riboses, and fragments were subjected to multiple rounds of a sequence consisting of NaIO_4_ oxidation treatments, subsequent removal of the 3′-end nucleotide, and de-phosphorylation. After several repetitions, an accumulation of Nm at the 3′-terminal position was to be expected. Positive enrichment of 3′-Nm-containing fragments, and thus improvement of Signal/noise ratio, is a very valuable feature for transcriptome-wide RNA analysis. However, the Nm-Seq protocol with repetitive oxidation/cleavage/de-phosphorylation cycles requires considerable amounts of input RNA, due to losses during such treatment.

Another recently established technique, named AlkAnilineSeq [74], relies on an alternative approach for selective fragment enrichment (Figure 2c). The method is based on the properties of RNA abasic sites, which are readily cleaved by aniline via β- and δ-elimination. These two consecutive reactions leave a 5′-phosphate at the N + 1 nucleotide in the sequence, which renders the corresponding fragments uniquely competent for subsequent adapter ligation. Thus, these abasic sites, which were created by alkaline hydrolysis at positions containing m^7^G and m^3^C, ultimately led to a positive selection of fragments resulting from modifications, and thus to exquisite sensitivity, because large populations of irrelevant RNA sequences were excluded from the library. This procedure, which was also shown to be partially sensitive to D and ho^5^C residues, thus required very limited amounts of input RNA and showed exceptional selectivity and specificity because of extremely low background.

The intrinsic reactivity of RNA (or DNA) abasic sites can be explored for detection of RNA modifications. For DNA, formation of abasic site is generally catalyzed by specific DNA-glycosylase, which removes damaged DNA bases, and subsequently the aldehyde group of abasic site can be chemically ligated to, e.g., a reactive amine, which in turn is coupled to biotin [75,76]. A similar approach was also proposed for the detection of oxidized sites in RNA [77,78]. Up to now, aldehyde reactivity at the RNA abasic site or decomposed RNA modified nucleotide was not yet explored for coupling to deep sequencing.

## 5. Conclusions

At first glance, this overview might convey the impression that RNA-Seq based detection methods can be successfully applied for mapping of almost any desired RNA modification throughout the transcriptome. However, while ever-new concepts for modification mapping get published, the community looks to the biology emerging, based on the methods published since 2012. Now, about six years after the furious resurrection of research on m^6^A in mRNA, that field is in full swing, with high impact papers appearing on a weekly basis, despite the fact that comprehensive mapping of m^6^A at single nucleotide resolution in a quantifiable manner is still a largely unresolved problem.

Detection of 2′-O-methylations in abundant RNAs like rRNA and tRNA is quite robust and is now applied even to quantitative analysis of RNA modification modulation under different conditions [79,80,81], but high-confidence transcriptome-wide 2′-O-Me screening has not yet been reported, even if some unpublished observations were made available to epitranscriptomic community (https://doi.org/10.1101/271916).

In contrast, although the performance, at least on paper, of published methods for the mapping of m^5^C and pseudouridine in mRNA are much better, papers on this topic issue from only a handful of research groups, and especially the numbers of reported modification sites have been met with skepticism [46,62,63]. Similarly, the occurrence of m^1^A in mRNA, as such, is agreed upon, but the opinions on numbers differ by two orders of magnitude [18,19].

Clearly, such controversies present a formidable obstacle for new groups to engage in targeted research on the effect of mRNA modifications. Research on the impact of defined modification sites is likely to focus on those sites identified by several labs in agreement. This strongly suggests that conservative “candidate-calling” might benefit the field. This means avoidance of false positives at the risk of overlooking some residues, i.e., producing false negatives out of caution.
